# Design, development and deployment of a web-based interoperable registry for inherited retinal dystrophies in Portugal: the IRD-PT

**DOI:** 10.1186/s13023-020-01591-6

**Published:** 2020-10-27

**Authors:** João Pedro Marques, Ana Luísa Carvalho, José Henriques, Joaquim Neto Murta, Jorge Saraiva, Rufino Silva

**Affiliations:** 1 Ophthalmology Unit, Centro de Responsabilidade Integrado em Oftalmologia (CRIO), Centro Hospitalar e Universitário de Coimbra (CHUC), Praceta Prof. Mota Pinto, 3000-075 Coimbra, Portugal; 2 University Clinic of Ophthalmology, Faculty of Medicine, University of Coimbra (FMUC), Coimbra, Portugal; 3 Clinical Academic Center of Coimbra (CACC), Coimbra, Portugal; 4 Medical Genetics Unit, Centro Hospitalar e Universitário de Coimbra (CHUC), Coimbra, Portugal; 5 University Clinic of Medical Genetics, Faculty of Medicine, University of Coimbra (FMUC), Coimbra, Portugal; 6 Instituto de Oftalmologia Dr. Gama Pinto (IOGP), Lisbon, Portugal; 7 University Clinic of Pediatrics, Faculty of Medicine, University of Coimbra (FMUC), Coimbra, Portugal

**Keywords:** Inherited retinal dystrophies, Registry, Rare diseases, Interoperability, Software, Data management, Research, Epidemiology, Natural history, Clinical genetics

## Abstract

**Background:**

The development of multicenter patient registries promotes the generation of scientific knowledge by using real-world data. A country-wide, web-based registry for inherited retinal dystrophies (IRDs) empowers patients and community organizations, while supporting formal partnerships research. We aim to describe the design, development and deployment of a country-wide, with investigators and stakeholders in the global aim to develop high-value, high-utility web-based, user-friendly and interoperable registry for IRDs—the IRD-PT.

**Results:**

The IRD-PT is a clinical/genetic research registry included in the *retina.pt* platform (https://www.retina.com.pt), which was developed by the Portuguese Retina Study Group. The *retina.pt* platform collects data on individuals diagnosed with retinal diseases, from several sites across Portugal, with over 1800 participants and over 30,000 consultations to date. The IRD-PT module interacts with the *retina.pt* core system which provides a range of basic functions for patient data management, while the IRD-PT module allows data capture for the specific purpose of IRDs. All IRDs are coded accordingly to the International Statistical Classification of Diseases and Related Health Problems (ICD) 9, ICD 10, ICD 11, and Orphanet Rare Disease Ontology (ORPHA codes) to make the IRD-PT interoperable with other IRD registries across the world. Furthermore, the genes are coded according to the Ontology of Genes and Genomes and Online Mendelian Inheritance in Man, whereas signs and symptoms are coded according to the Human Phenotype Ontology. The IRD-PT module pre-launched at *Centro Hospitalar e Universitário de Coimbra*, the largest reference center for IRDs in Portugal. As of April 1st 2020, finalized data from 537 participants were available for this preliminary analysis.

**Conclusions:**

In the specific field of rare diseases, the use of registries increases research accessibility for individuals, while providing clinicians/investigators with a coherent data ecosystem necessary to boost research. Appropriate design and implementation of patient registries enables rapid decision making and ongoing data mining, ultimately leading to improved patient outcomes. We have described here the principles behind the design, development and deployment of a web-based, user-friendly and interoperable software tool aimed to generate important knowledge and collecting high-quality data on the epidemiology, genomic landscape and natural history of IRDs in Portugal.

## Background

The Agency for Healthcare Research and Quality defines a registry as “an organized system that uses observational study methods to collect uniform data (clinical and other) to evaluate specified outcomes for a population defined by a particular disease, condition, or exposure, and that serves one or more predetermined scientific, clinical, or policy purposes” [1]. Clinical registries have existed for decades in the field of ophthalmology [2–5], serving a variety of purposes, which include (1) capturing the epidemiologic features of an ocular disease or condition, (2) tracking outcomes and complications of drugs or procedures, (3) recording adverse events, or (4) combinations of the above [6]. In recent years, policy makers started recognizing clinical registries as an important tool for improving the value of healthcare. Outcome data is now used to fill in gaps of evidence that cannot be provided by randomized controlled trials [6]. Furthermore, data from clinical registries is also increasingly being used to facilitate learning networks and to establish research collaborations between scientific researchers, clinicians, industry, regulators, patient organizations, patients and families [7]. This is especially true for rare diseases where the small number of cases for each disease creates additional barriers in the translational research pathway, and makes identification and establishment of a substantial cohort a very difficult task.

Inherited retinal dystrophies (IRDs) are a clinically and genetically heterogenous group of diseases with an estimated prevalence of 1 in 3000 individuals [8]. Despite some common ground, genetic profiles vary considerably among regions and ethnic groups [9–16], thus highlighting the importance of obtaining reference population-based data. The presence of founder mutations may greatly contribute for these differences, as observed in a large Israeli population [9]. While local hospital-based registries may provide high quality information and resources, their coverage is usually small. To fully understand the prevalence and genomic landscape of IRDs, we must connect knowledge that is widespread throughout miscellaneous registries. The development of multicenter patient registries and natural history studies promote the generation of scientific knowledge by using real-world data. As rare diseases gain visibility as a public health priority and the marketplace expands, acknowledgement of the importance of building collaborative relationships in rare disease research increases [7]. A national, web-based registry for IRDs is able to empower patients and community organizations, while supporting formal partnerships with investigators and stakeholders in the global aim to develop high-value, high-utility research.

When developing a registry, it is essential to ensure that it is ethically governed, user-friendly and designed with maximum sustainability. This includes the implementation of foundational, structural, semantic, and organizational interoperability processes to optimize the utility of data and allow its linkage to other existing or future registries [7]. By making data computationally accessible, it is possible to bridge compatibility gaps between different hospitals, healthcare systems, registries and languages [17]. Adoption of comprehensive phenotype and rare disease ontologies enables this type of sharing by making data findable, accessible, interoperable, and re-usable (FAIR principles) [18]. These features have made Orphanet Rare Disease Ontology (ORDO) a standard for rare disease coding in European health-care systems and led to the widespread adoption of ontologies like the Human Phenotype Ontology (HPO) by global genomics initiatives, like the European Reference Network for Rare Eye Disease (ERN-EYE) [17].

The purpose of this study is to describe the design, development and deployment of a country-wide, web-based, user-friendly and interoperable registry for IRDs—the IRD-PT.

## Results

### Data capture

The IRD-PT was designed to capture longitudinal data on IRDs. The data captured by the IRD-PT module is kept to a minimum to deliver an efficient and user-friendly data collecting tool. The user must complete all the mandatory fields/check all the mandatory boxes in order to save the entry. However, the system allows editing and/or completion of previously unanswered non-mandatory fields at the user’s convenience. The list of covered clinical diagnoses is shown on Table 1, while the list of the genes and their respective Ontology of Genes and Genomes (OGG) and Mendelian Inheritance in Man (MIM) numbers are shown on Table 2. Even though inherited optic neuropathies and other genetically-associated retinal diseases (such as Pseudoxanthoma Elasticum-associated retinopathy or isolated foveal hypoplasia) are not IRDs per se, we opted to include them in the registry since these are common diagnoses in an Ophthalmic Genetics clinic. This is not something previously unseen. In fact, these diseases are also part of the Inherited Retinal Disease Classification proposed by Stone et al. [16].

**Table 1:** List and ORPHA numbers of the clinical diagnoses covered by the IRD-PT module

	Inherited retinal dystrophies^a^	
	ORPHA 71862	
**1. ISOLATED PROGRESSIVE INHERITED RETINAL DISORDER (ORPHA 519306)**	**3. SYNDROMIC INHERITED RETINAL DISORDER (ORPHA 519325)**	**5. CHORIORETINAL DYSTROPHIES (ORPHA 519,300)**
1.1 Retinitis punctata albescens (ORPHA 52427)	3.1. Alström syndrome (ORPHA 64)	5.1. Bietti crystalline dystrophy (ORPHA 41751)
1.2. ARB (ORPHA 139455)	3.2. Jalili syndrome (ORPHA 1873)	5.2. CACD (ORPHA 75377)
1.3. Cone/cone-rod dystrophy (ORPHA 1872)	3.3. Senior-Loken syndrome (ORPHA 3156)	5.3. Choroideremia (ORPHA 180)
1.4. Late-onset retinal degeneration (ORPHA 67042)	3.4. Joubert syndrome (ORPHA 475)	5.4. Gyrate atrophy of choroid and retina (ORPHA 414)
1.5. Leber congenital amaurosis (ORPHA 65)	3.5. Usher syndrome (ORPHA 886)	5.5. Helicoid peripapillary chorioretinal degeneration (ORPHA 86813)
1.6. Retinitis Pigmentosa AR (ORPHA 791)	3.6. Bardet-Biedl syndrome (ORPHA 110)	5.6. Pigmented paravenous retinochoroidal atrophy (ORPHA 251295)
1.7. Retinitis Pigmentosa AD (ORPHA 791)	3.7. Hallervorden-Spatz syndrome (ORPHA 157850)	**6. HEREDITARY OPTIC NEUROPATHY (ORPHA 98671)**
1.8. Retinitis pigmentosa XL (ORPHA 791)	3.8. Syndromic retinitis pigmentosa—other (ORPHA 519325)	6.1. Autosomal dominant optical atrophy (ORPHA 98672)
**1.9. Isolated macular dystrophy (ORPHA 519302)**	3.9. Kearns-Sayre syndrome (ORPHA 480)	6.2. Leber hereditary optic atrophy (ORPHA 104)
1.9.1. Sorsby fundus dystrophy (59181)	3.10. PXE (ORPHA 758)	6.3. Hereditary optic neuropathy—other (98671)
1.9.2. Stargardt disease (ORPHA 827)	3.11. Alport Syndrome (ORPHA 63)	
1.9.3. Best vitelliform macular dystrophy (ORPHA 1243)	3.12. MIDD (ORPHA 225)	**7. OTHER RARE DISORDERS OF THE POSTERIOR SEGMENT OF THE EYE (ORPHA 519311)**
1.9.4. North Carolina macular dystrophy (ORPHA 75327)	3.13. Cuticular drusen/C3 Glomerulopathy (ORPHA 329918)	7.1. Foveal hypoplasia (ORPHA 519398)
**1.10. Pattern dystrophy (ORPHA 63454)**	**4. INHERITED VITREOUS DYSTROPHIES (ORPHA 519304)**	7.2. Coloboma (ORPHA 98942)
1.10.1. Butterfly-shaped pigment dystrophy (ORPHA 99001)	4.1. X-linked retinoschisis (ORPHA 792)	7.3. Ocular albinism (ORPHA 284804)
1.10.2. MFD simulating fundus flavimaculatus (ORPHA 99003)	4.2. Stickler syndrome (ORPHA 828)	7.4. Oculocutaneous albinism (ORPHA 55)
1.10.3. Reticular dystrophy of the RPE (ORPHA 99002)	4.3. Wagner disease (ORPHA 898)	7.5. Other
1.10.4. AOFVD (ORPHA 99000)	4.4. FFEVR (OPRHA 891)	
**2. ISOLATED STATIONARY INHERITED RETINAL DISORDER (ORPHA 519319)**	4.5. Goldmann-Favre syndrome/ESCS	
2.1. Achromatopsia (ORPHA 49382)	4.6. ADVIRC (ORPHA 3086)	
2.2. CSNB (ORPHA 215)		
2.3. Fundus albipunctatus (ORPHA 227796)		
2.4. Familial drusen/Malattia leventinese (ORPHA 75376)		

Bold corresponds to items (groups of diseases) that have dependences

*MFD* multifocal pattern dystrophy, *AOFVD* adult-onset foveomacular vitelliform dystrophy, *CSNB* congenital stationary night blindness, *PXE* pseudoxanthoma elasticum, *MIDD* maternally-inherited diabetes and deafness, *FEVR* familial exudative vitreoretinopathy, *ADVIRC* autosomal-dominant vitreoretinochoroidopathy, *CACD* central areolar choroidal dystrophy

^a^The platform allows the selection of more than one diagnosis

**Table 2:** List of available IRD genes^a^ and their respective Ontology of Genes and Genomes (OGG) and Mendelian Inheritance in Man (MIM) numbers

ABCA4	OGG:3000000024	MIM:601691	LRAT	OGG:3000009227	MIM:604863
ABCC6	OGG:3000000368	MIM:603234	MAK	OGG:3000004117	MIM:154235
ADGRV1	OGG:3000084059	MIM:602851	MERTK	OGG:3000010461	MIM:604705
AIPL1	OGG:3000023746	MIM:604392	MT-ND1	OGG:3000004535	MIM:516000
ALMS1	OGG:3000007840	MIM:606844	MT-ND4	OGG:3000004538	MIM:516003
BBS1	OGG:3000000582	MIM:209901	MT-ND4L	OGG:3000004539	MIM:516004
BBS10	OGG:3000079738	MIM:610148	MT-ND6	OGG:3000004541	MIM:516006
BBS12	OGG:3000166379	MIM:610683	MT-TL1	OGG:3000004567	MIM:590050
BBS2	OGG:3000000583	MIM:606151	MYO7A	OGG:3000004647	MIM:276903
BBS3/ARL6	OGG:3000084100	MIM:608845	NMNAT1	OGG:3000064802	MIM:608700
BBS4	OGG:3000000585	MIM:600374	NR2E3	OGG:3000010002	MIM:604485
BBS5	OGG:3000129880	MIM:603650	NRL	OGG:3000004901	MIM:162080
BBS7	OGG:3000055212	MIM:607590	NYX	OGG:3000060506	MIM:300278
BBS9	OGG:3000027241	MIM:607968	OAT	OGG:3000004942	MIM:613349
BEST1	OGG:3000007439	MIM:607854	OPA1	OGG:3000004976	MIM:605290
C1QTNF5	OGG:3000114902	MIM:608752	OPN1LW	OGG:3000005956	MIM:300822
CACNA1F	OGG:3000000778	MIM:300110	PANK2	OGG:3000080025	MIM:606157
CDH23	OGG:3000064072	MIM:605516	PAX6	OGG:3000005080	MIM:607108
CEP290	OGG:3000080184	MIM:610142	PCARE	OGG:3000388939	MIM:613425
CERKL	OGG:3000001399	MIM:608381	PDE6A	OGG:3000005145	MIM:180071
CFH	OGG:3000003075	MIM:134370	PDE6B	OGG:3000005158	MIM:180072
CHM	OGG:3000001121	MIM:300390	PDE6C	OGG:3000005146	MIM:600827
CLN3	OGG:3000001201	MIM:607042	PDE6G	OGG:3000005148	MIM:180073
CLRN1	OGG:3000007401	MIM:606397	PHYH	OGG:3000005264	MIM:602026
CNGA3	OGG:3000001261	MIM:600053	POC1B	OGG:3000282809	MIM:614784
CNGB1	OGG:3000001258	MIM:600724	PRCD	OGG:3000768206	MIM:610598
CNGB3	OGG:3000054714	MIM:605080	PROM1	OGG:3000008842	MIM:604365
CNNM4	OGG:3000026504	MIM:607805	PRPF3	OGG:3000009129	MIM:607301
COL2A1	OGG:3000001280	MIM:120140	PRPF31	OGG:3000026121	MIM:606419
COL4A3	OGG:3000001285	MIM:120070	PRPF8	OGG:3000010594	MIM:607300
COL4A4	OGG:3000001286	MIM:120131	PRPH2 (RDS)	OGG:3000005961	MIM:179605
COL4A5	OGG:3000001287	MIM:303630	RDH12	OGG:3000145226	MIM:608830
CRB1	OGG:3000023418	MIM:604210	RDH5	OGG:3000005959	MIM:601617
CRX	OGG:3000001406	MIM:602225	RHO	OGG:3000006010	MIM:180380
CYP4V2	OGG:3000285440	MIM:608614	RIMS1	OGG:3000022999	MIM:606629
DHDDS	OGG:3000079947	MIM:608172	RLBP1	OGG:3000006017	MIM:180090
EFEMP1	OGG:3000002202	MIM:601548	RP1	OGG:3000006101	MIM:3937
ELOVL4	OGG:3000006785	MIM:605512	RP2	OGG:3000006102	MIM:300757
EYS	OGG:3000346007	MIM:612424	RPE65	OGG:3000006121	MIM:180069
FAM161A	OGG:3000084140	MIM:613596	RPGR	OGG:3000006103	MIM:312610
GNAT1	OGG:3000002779	MIM:139330	RPGRIP1	OGG:3000057096	MIM:605446
GNAT2	OGG:3000002780	MIM:139340	RS1	OGG:3000006247	MIM:300839
GPR98	OGG:3000084059	MIM:602851	SAG	OGG:3000006295	MIM:181031
GRK1	OGG:3000006011	MIM:180381	SEMA4A	OGG:3000064218	MIM:607292
GUCA1A	OGG:3000002978	MIM:600364	SNRNP200	OGG:3000023020	MIM:601664
GUCA1B	OGG:3000002979	MIM:602275	SPATA7	OGG:3000055812	MIM:609868
GUCY2D	OGG:3000003000	MIM:600179	TIMP3	OGG:3000007078	MIM:188826
HGSNAT	OGG:3000138050	MIM:610453	TOPORS	OGG:3000010210	MIM:609507
IMPDH1 (RP10)	OGG:3000003614	MIM:146690	TULP1	OGG:3000007287	MIM:602280
IMPG1	OGG:3000003617	MIM:602870	USH1G	OGG:3000124590	MIM:607696
IMPG2	OGG:3000050939	MIM:607056	USH2A	OGG:3000007399	MIM:608400
IQCB1	OGG:3000009657	MIM:609237	VCAN	OGG:3000001462	MIM:118661
KCNV2	OGG:3000169522	MIM:607604	WDR19	OGG:3000057728	MIM:608151
KLHL7	OGG:3000055975	MIM:11119	Other	N/A	N/A
LCA5	OGG:3000167691	MIM:611408	Inconclusive	N/A	N/A

^a^The user may select one, two or more genes in case clinically relevant variants are found in more than one gene. This list may be edited with newer additions in case other genes are found in the Portuguese population with IRDs

We were able to design an interoperable module by reusing the *retina.pt* core data elements where appropriate (epidemiological data such as sex, date of birth and patient ID), whilst also incorporating bespoke data elements, sections and forms for the specific field of IRDs (Table 3). Upon selection of a particular item (clinical diagnosis, signs and symptoms, syndromic features, gene or additional diagnoses), a hyperlink is available to direct the user to the correspondent ontology webpage (ORPHA, HPO, OGG).

**Table 3:** Data set for the IRD-PT module, including the Human Phenotype Oncology (HPO) coding when applicable

Field	Type of entry	Answer	Available options
1. Patient ID	Free text	Mandatory	
2. Date of birth	Date format	Mandatory	
3. Sex	Select from list	Mandatory	Male; female
4. Date of diagnosis	Date format	Mandatory	
5. Clinical diagnosis	Select from list (allows selection of more than one option)	Mandatory	See Table 1
6. Consanguinity	Select from list	Mandatory	Yes; no; suspected
7. Family history	Select from list	Mandatory	Yes; no; suspected
7.1. Family linkage section (only shows if the user answered Yes to the previous question)	Allows introduction of one or multiple affected family members, including their family relation to the patient (brother; sister; mother; father; son; daughter; uncle; aunt; cousin; grandfather; grandmother; other) and Hospital ID which has a hyperlink to that patient’s page in case he/she has consented to be part of the registry		
8. Signs and symptoms	Select from list (allows selection of more than one option)	Mandatory	Nyctalopia (HP:0000662); decreased VA (HP:0000529); photophobia (HP:0000613); color vision defects (HP:0000551); central scotoma (HP:0000603); constricted visual field (HP:0001133); photopsia (HP:0030786); nystagmus (HP:0,000,639); headache (HP:0002315); migraine (HP:0002076); visual hallucinations (HP:0002367); other
9. Age of onset of symptoms	Select from list	Mandatory	at birth; < 5; 6–10; 11–20; 21–30; 31–50; > 51
10. Syndromic features	Select from list	Mandatory	Yes/no
10.1. Syndromic features list (only shows if the user answered yes to the previous question)	Select from list (allows selection of more than one option)	Optional	Hearing loss/deafness (HP:0008527); obesity (HP:0001513); hypogonadism (HP:0000135); diabetes mellitus (HP:0000819); diabetes insipidus (HP:0000873); polydactyly (HP:0010442); other skeletal abnormalities (HP:0000924); cognitive impairment (HP:0100543); developmental delay (HP:0001263); seizures (HP:0001250); ataxia (HP:0001251); dysarthria (HP:0001260); renal insufficiency (HP:0000083); other
11. Genetic testing	Select from list	Mandatory	Yes/no
11.1. Type of test (only shows if the user answered yes to genetic testing)	Select from list (allows selection of more than one option)	Optional	Sanger sequencing; NGS panel; WES; MLPA; don’t know; other
11.2. Gene (only shows if the user answered yes to genetic testing)	Select from list (allows selection of more than one option)	Optional	See Table 2
11.3. Variants^a^ (only shows if the user answered yes to genetic testing)	Free text	Optional	
11.4. Classification of variants (ACMG) (only shows if the user answered yes to genetic testing)	Select from list (for each introduced variant)	Optional	Pathogenic; likely pathogenic; VUS
BCVA^b^	Select from list	Mandatory	From 20/1000 to 20/10
IOP^b^	Free text	Optional	Only accepts numbers from 01 to 99
Additional diagnoses^b^	Select from list (allows selection of more than one option)	Optional	Amblyopia (HP:0000646); cataract (HP:0000518); CNV (HP:0011506); CME (HP:0011505); glaucoma (HP:0000501); ERM (HP:0100014); macular hole (HP:0011508); lamellar hole (HP:0001103); macular pseudohole (HP:0001103); vitreomacular traction (HP:0031151); retinal detachment (HP:0000541); keratoconus (HP:0000563); strabismus (HP:0000486); other
Previous treatments^b^	Select from list (allows selection of more than one option)	Optional	Vitreoretinal surgery; strabismus surgery; glaucoma surgery; YAG laser capsulotomy; corneal transplant; cataract surgery; intravitreal injection; subretinal injection; laser photocoagulation; refractive surgery; other

*DOB* date of birth, *NGS* next generation sequencing, *WES* whole exome sequencing, *MLPA* multiplex ligation-dependent probe amplification, *ACMG* American College of Medical Genetics and Genomics, *VUS* variant of uncertain significance, *BCVA* best corrected visual acuity, *IOP* intraocular pressure, *CNV* choroidal neovascularization, *CME* cystoid macular edema, *ERM* epiretinal membrane

^a^Apart from listing the variants as free text, an icon is available for the upload of the raw sequencing file

^b^These fields appear separately for the right and left eye

The family linkage section allows simple viewing of the details of affected family members that are also part of the registry. At the end of each visit, a free text area is available for comments (follow-up, imaging, prescription, etc.).

Longitudinal data is captured through specific follow-up forms. The platform allows retrospective data introduction. As the program develops, and through alignment with international data collection for IRD clinical registries, the IRD-PT core data set may be modified or extended to include additional key clinical variables.

### Data analysis and graphical displays

**Fig. 1:**
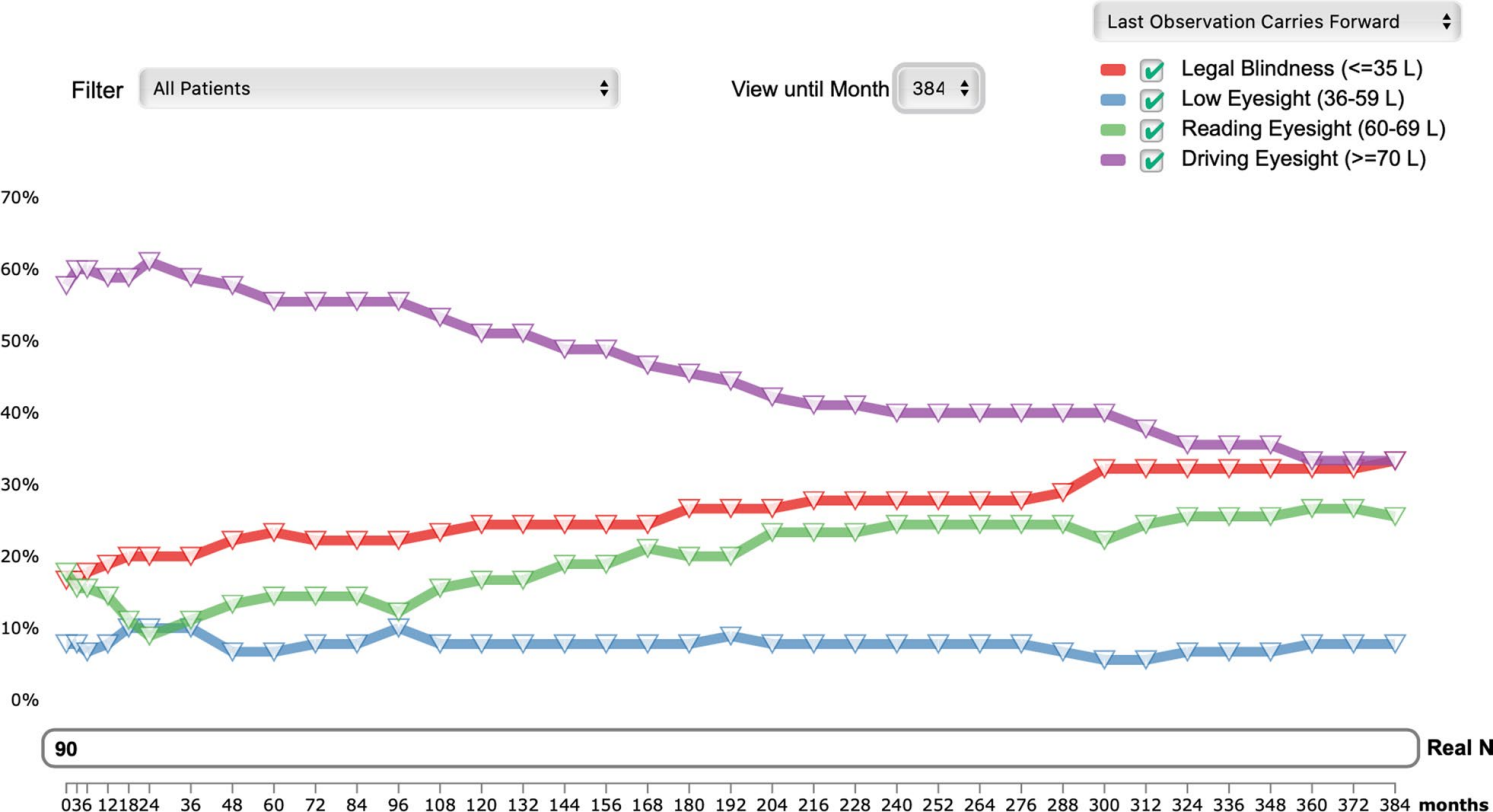
Variation in the percentage of eyes with different levels of BCVA (ETDRS letters) over time (last-observation carried forward) in the 45 patients (90 eyes) with Usher syndrome included in the IRD-PT registry so far. The graph is automatically provided by the platform. The user may select which parameters to show. It is also possible to select only one eye per patient

**Fig. 2:**
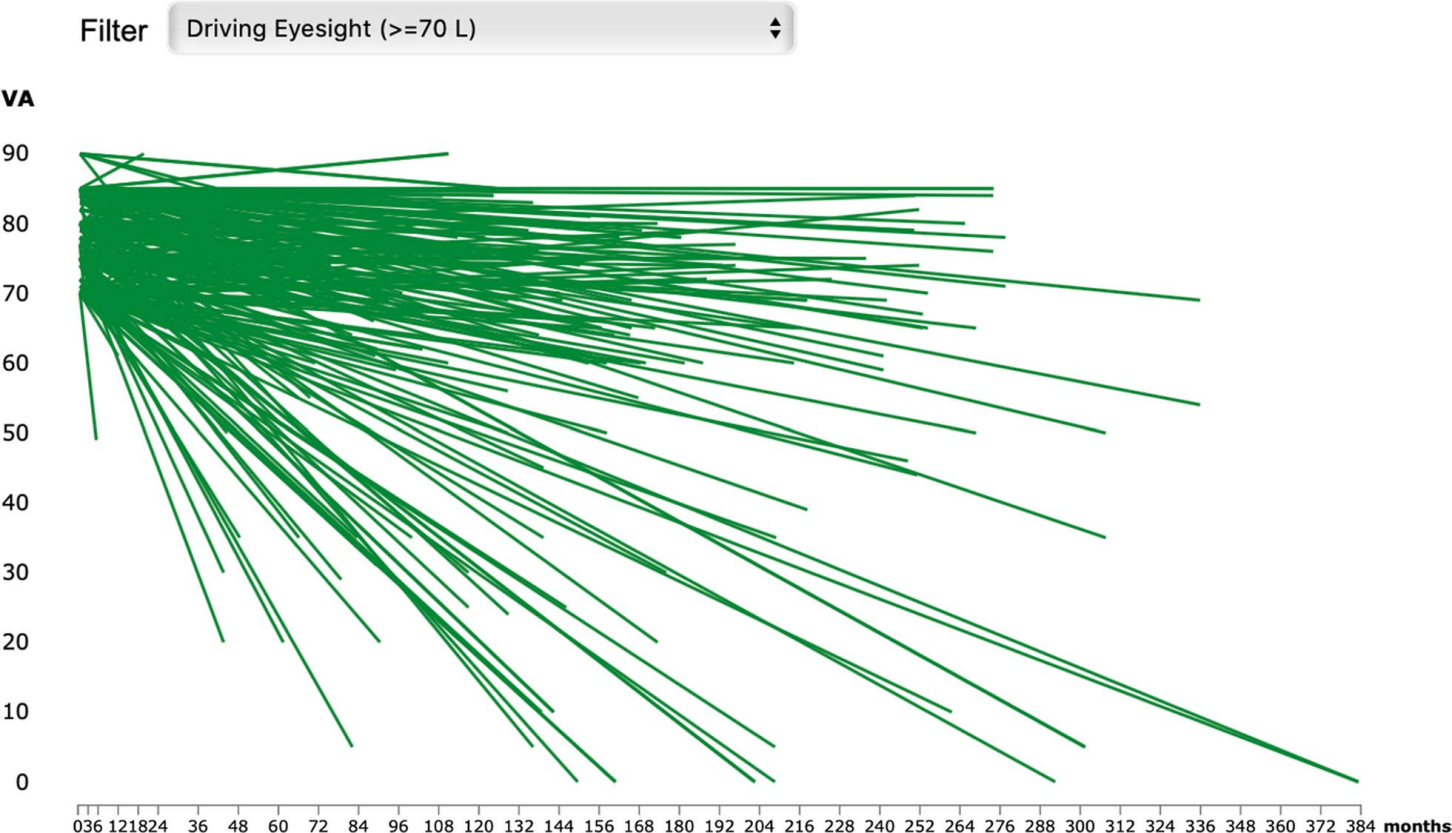
Progression of BCVA (ETDRS letters) over follow-up (last-observation carried forward) in eyes with any IRD that started with driving vision (≥ 70 ETDRS letters). Each green line corresponds to an eye of an individual patient. The graph is automatically provided by the platform. The user may select which parameters to show

Since the *retina.pt* was designed to be both a registry and a research tool, data export and analysis features are very important. A search engine that allows data filtering is available for the user to search specific anonymized data, such as the total number of affected patients or the total number of affected families with a certain disease-causing gene, clinical diagnosis, BCVA level, etc. Furthermore, the platform offers statistical tools for simple analyses and these are also available for the IRD-PT module (Figs. 1, 2). For more sophisticated analyses, users can export their own data on *excel* format and analyze it as they see fit. Data are aggregated in an anonymized fashion, without identification of the individual patients.

### Participant characteristics

**Fig. 3:**
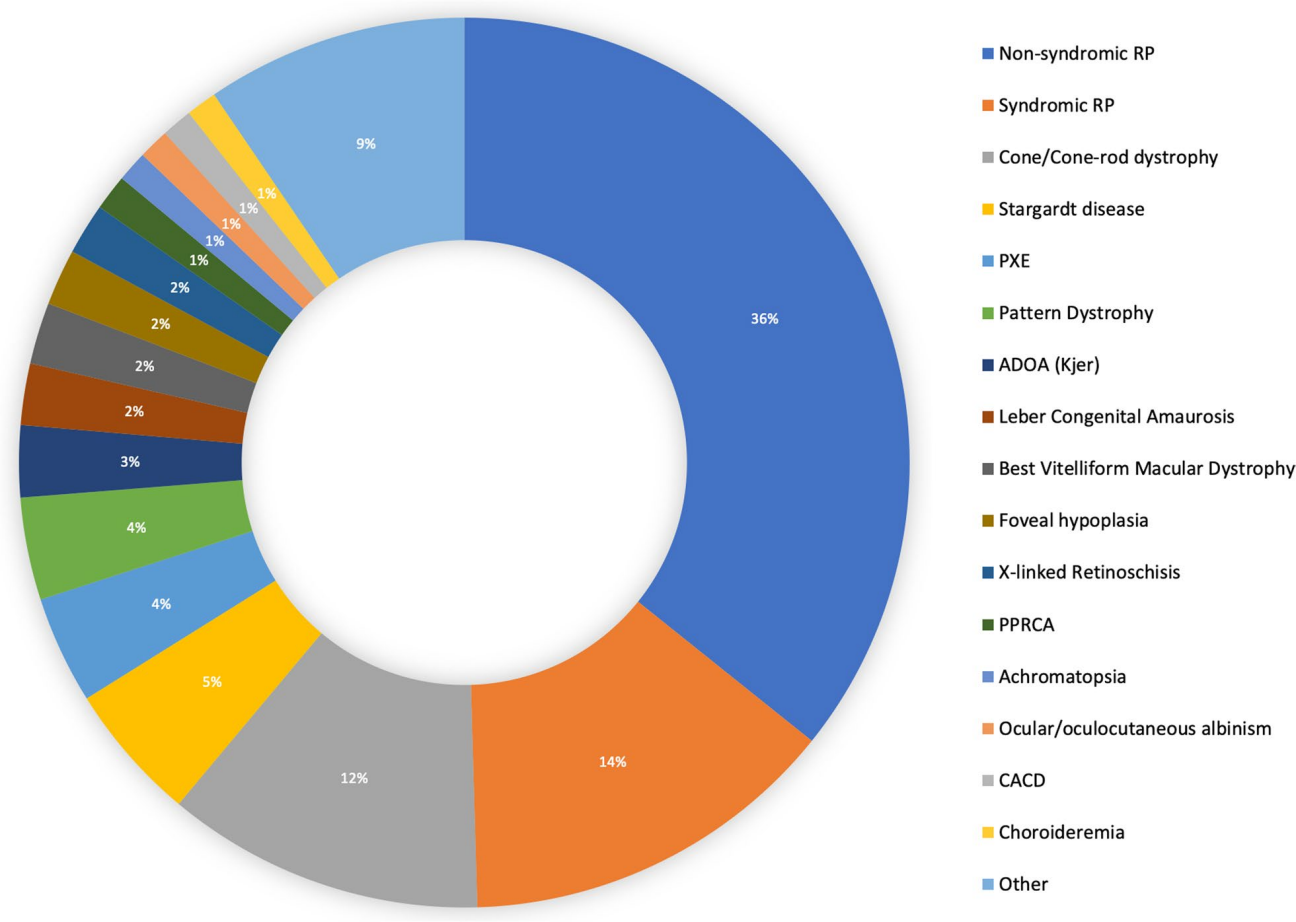
Graphical representation of the relative frequency of each clinical diagnosis in the 537 patients included in the IRD-PT registry. Those with < 1% cases are expressed under the tag *Other*. *RP* retinitis pigmentosa, *PXE* pseudoxanthoma elasticum, *ADOA* autosomal dominant optic atrophy, *PPRCA* pigmented paravenous retinochoroidal atrophy, *CACD* central areolar choroidal dystrophy

**Fig. 4:**
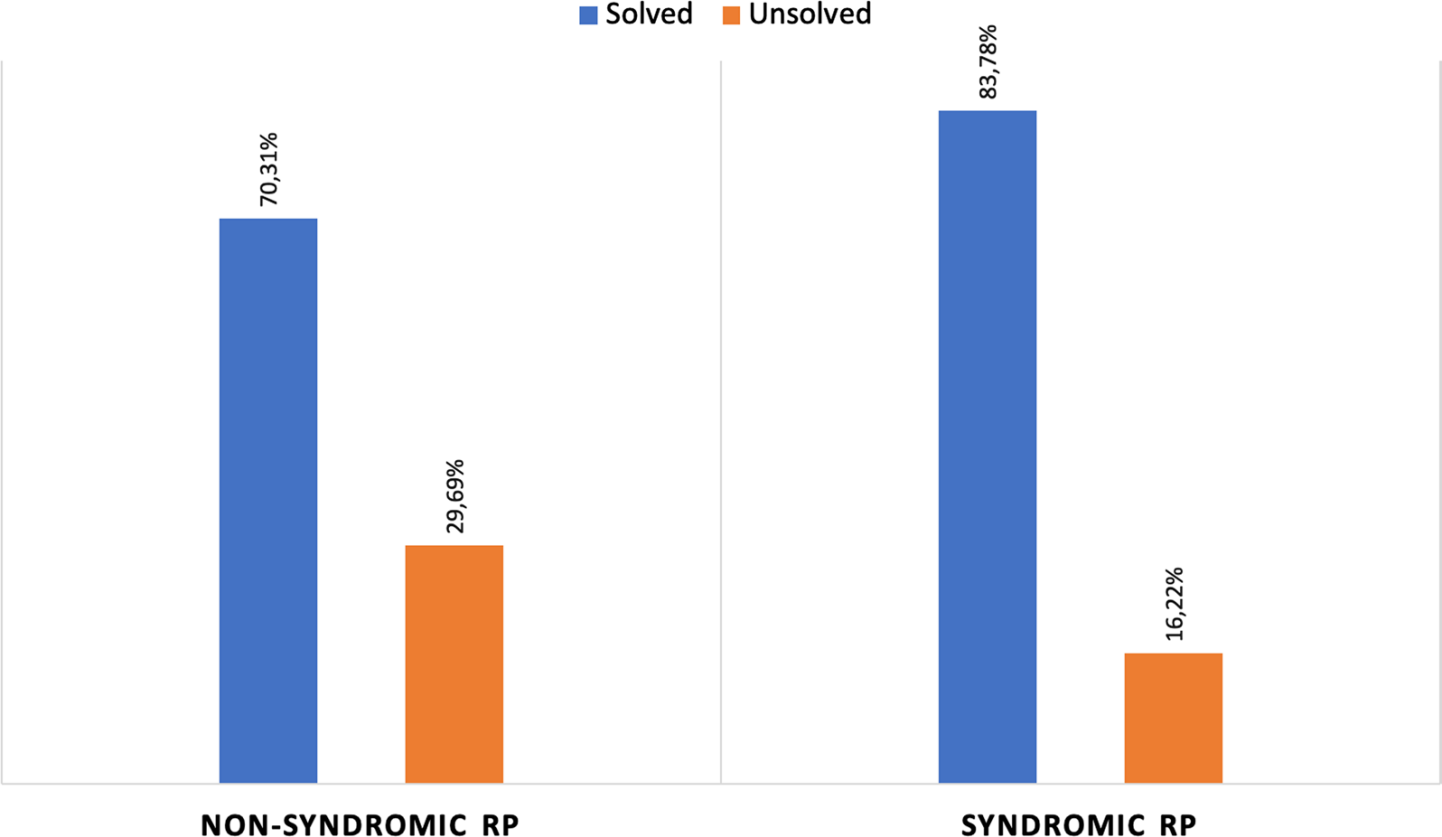
Graphical representation of the percentage of genetically solved and genetically unsolved cases of syndromic and non-syndromic retinitis pigmentosa in our cohort

So far, the *retina.pt* platform has been approved by the Human Research Ethics Committee (HREC)/Institutional Review Board (IRB) of 52 health care providers across Portugal. Each of these hospitals/clinics has established the necessary infrastructure to support rapid rollout of site and patient recruitment, data collection, and data transfer. One-hundred and thirty five users (doctors/investigators) have applied for credentials to access the registry, and 58 of these have already included patient data. To date, there are over 1800 participants (patients) and over 30,000 consultations included in the registry. In mid 2019, the IRD-PT module was pre-launched at *Centro Hospitalar e Universitário de Coimbra* (CHUC), the only Portuguese health care provider (HCP) that is a member of the ERN-EYE, and the largest reference center for IRDs in Portugal. The idea of testing the registry in one dedicated center before its national debut was aimed to identify possible problems during data completion, test the time spent in data entry, and detect information gaps or system inaccuracies. The registry proved fully functional, fast and easy to use. As of April 1st 2020, finalized data from 537 participants were available for this preliminary analysis. Considering the Portuguese population (~ 10 million inhabitants), this number corresponds to roughly 1/6 of the total estimated cases of IRDs in Portugal. The distribution of the clinical diagnoses and their relative frequency among the included participants is shown in Table 4. As illustrated in Fig. 3, syndromic (14%) and non-syndromic retinitis pigmentosa (36%) account for 50% of the clinical diagnoses. The percentage of genetically solved and unsolved cases of syndromic and non-syndromic RP is shown in Fig. 4. Of all participants included in the IRD-PT registry to date, 57% are women and the mean age at the index visit was 39.27 ± 19.03 years. Average baseline BCVA was 54.36 ± 27.22 and final BCVA was 47.64 ± 28.92 ETDRS letters.

**Table 4:** Distribution of the clinical IRD diagnoses and their relative frequency among the 537 subjects included in the IRD-PT registry

Clinical diagnosis	n	Relative frequency (%)
Non-syndromic RP	192	35.75
Syndromic RP	74	13.78
Cone/cone-rod dystrophy	62	11.55
Stargardt disease	27	5.03
PXE	21	3.91
Pattern dystrophy	20	3.72
ADOA (Kjer)	14	2.61
Leber congenital amaurosis	12	2.23
Best vitelliform macular dystrophy	12	2.23
Foveal hypoplasia	11	2.05
X-linked retinoschisis	10	1.86
PPRCA	7	1.30
Achromatopsia	6	1.12
Ocular/oculocutaneous albinism	6	1.12
CACD	6	1.12
Choroideremia	6	1.12
CSNB	5	0.93
Coloboma	5	0.93
ARB	4	0.74
Bietti crystalline dystrophy	4	0.74
Fundus albipunctatus	4	0.74
MIDD	4	0.74
Gyrate atrophy of choroid and retina	3	0.56
Goldmann-Favre syndrome/ESCS	3	0.56
Stickler/Wagner syndrome	3	0.56
Cuticular drusen/C3 glomerulopathy	3	0.56
LORD	3	0.56
LHON	3	0.56
ADVIRC	2	0.37
Retinitis punctata albescens	2	0.37
Alport syndrome	2	0.37
NCMD	1	0.19

*RP* retinitis pigmentosa, *PXE* pseudoxanthoma elasticum, *ADOA* autosomal dominant optic atrophy, *PPRCA* pigmented paravenous retinochoroidal atrophy, *CACD* central areolar choroidal dystrophy, *CSNB* congenital stationary night blindness, *ARB* autosomal recessive bestrophinopathy, *MIDD* maternally inherited diabetes and deafness, *ESCS* enhanced S-cone syndrome, *LORD* late-onset retinal degeneration, *LHON* leber hereditary optic neuropathy, *ADVIRC* autosomal dominant vitreoretinochoroidopathy, *NCMD* North Carolina macular dystrophy

## Discussion

Appropriate design, implementation and deployment of patient registries enables rapid decision making and ongoing data mining, ultimately leading to improved patient outcomes [7, 19, 20]. In the specific field of rare diseases, the use of registries increases research accessibility for individuals, while providing clinicians/investigators with a coherent data ecosystem necessary to boost research. The IRD-PT module of *retina.pt* will facilitate the efficient capture of accurate, longitudinal, country-wide data for IRDs. The registry will provide valuable information on disease prevalence, genomic landscape, genotype–phenotype correlations and natural history of IRDs, which is currently an unmet need in Portugal. Furthermore, the registry will facilitate patient selection for newly approved treatments or enrollment in clinical trials. The use of a web-based data storage system allows the registry to extend recruitment across multiple centers in the country. The modular design and scalable nature of the framework used to deploy the IRD-PT registry make it easily adaptable over time, ensuring its long-term sustainability. Furthermore, the use of domain-specific ontologies adds value to data, through an integrated knowledge base that is searchable and comparable by user and by machines [17, 21]. In fact, by resorting to common data elements, core outcome sets, and standardized data structures, the IRD-PT module can support the exchange of data across datasets, facilitating the connection to other registries at an international level. The interoperability of this registry by means of data harmonization is a key feature pointing to its utility and scalability. Another important issue of a web-based registry is usability, i.e. the capacity of a software system to provide conditions for its users to perform the tasks satisfactorily, effectively, and efficiently. Ophthalmologists have limited time with patients during office visits, and electronic health record (EHR) use requires a substantial portion of that time, therefore affecting productivity [22, 23]. The *retina.pt* registry combines a user-friendly platform and reduced load of data entry with the possibility to generate a *pdf* document that can be saved, printed or copied to the hospital EHR system, thus eliminating the need for duplicate records. Additionally, there is also the possibility of EHR third party applications with structured information to deliver their data directly to specific subfields of the registry, thus enabling a quick fill in process. The detailed information provided on Table 3 regarding data capture for the IRD-PT may be used to modify EHR systems to allow for direct data transfer. Finally, the versatility of the platform, makes it possible to serve as electronic case report form (eCRF) for upcoming observational, natural-history or post-market authorization studies.

The IRD-PT is not exempt of limitations. An important principle in registry design is to reduce the load of data entry. This does not come without a price. By limiting the data that is considered mandatory to a minimum, there may be incomplete information/missing data for some included subjects concerning unanswered non-mandatory fields. Another limitation is that grading systems/levels for the symptoms or degree of impairment are not available. The fact that symptoms are simply marked as present/not present prevents a precise characterization of these symptoms during the disease course. Finally, since each user is responsible for its own data entry, we cannot be sure about the accuracy of its contents. This may be particularly problematic when a case is considered molecularly solved or unsolved. Misinterpretation of the genetic findings is not uncommon, which may lead to selection bias regarding the number of molecularly solved/unsolved cases.

## Conclusions

We have described here the principles behind the design, development and deployment of a web-based software tool that forms the basis of a nation-wide registry for IRDs. The pre-launch of the IRD-PT module in the largest Portuguese referral center for IRDs (CHUC), allowed to test the functionalities of the registry and enroll the first 537 IRD patients, roughly 1/6 of the total estimated cases of IRDs in Portugal. Now that the module is fully working, recruitment will be extended to other Portuguese hospitals. Judging from the enthusiasm and adherence observed with the launch of the *retina.pt* platform, we believe that the IRD-PT registry will be rapidly adopted by the Portuguese ophthalmologists managing IRD patients. Our hope is to generate important knowledge and collect high-quality data on the epidemiology, genomic landscape, genotype–phenotype correlations and natural history of IRDs in Portugal. This will both boost and excel clinical research in the field of IRDs in our country, while facilitating patient access to clinical trials or new therapies.

## Methods

### Registry design

The IRD-PT is a clinical/genetic research registry. Its main goal is to create a national, web-based registry of IRDs in Portugal that allows to study their prevalence, genomic profile, genotype–phenotype correlations and natural history. Also, the registry may assist in the recruitment of participants for new treatments/clinical trials, and provide support for the establishment of disease-specific standards and care. The IRD-PT registry is included in the *retina.pt* platform (https://www.retina.com.pt), which was developed by the Portuguese Retina Study Group (GER, www.ger-portugal.com). The *retina.pt* registry deployed in 2017 to fulfil a vital component on patient-centered care for retinal diseases. It collects data on individuals diagnosed with retinal diseases, from several sites across Portugal, with over 1800 participants and over 30,000 consultations to date. The IRD-PT is a module interacting with the *retina.pt* core system. The core system provides a range of basic functions used for patient data management, while the IRD-PT module provides the user with the functionality to capture data for the specific purpose of IRDs.

### Recruitment and informed consent

Both pediatric and adult patients with a genetic and/or clinical diagnosis of IRD living in Portugal and attending Ophthalmology clinics around the country are invited to participate. Participation in the registry is voluntary. Before enrollment, the participant (patient) or their legally authorized representative must provide informed consent for the collection, storage, and use of their personal health data. No costs or compensations are involved for participants or their family members as the data collected in the IRD-PT module refers to information routinely collected by the responsible physician. All included subjects are allowed to withdraw their consent at any time, without providing a reason. This does not impact their regular follow-up at the clinic.

### Ethics and regulations

The registry meets the necessary requirements for compliance with the General Data Protection Regulation (GDPR) of the European Union and all approvals were obtained prior to recruiting patients for the registry. Formal review and approval was obtained from the Portuguese Data Protection Authority (*Comissão Nacional de Proteção de Dados*—CNPD), HREC of *Centro Hospitalar e Universitário de Coimbra* (CHUC) and IRB of the Faculty of Medicine of the University of Coimbra (FMUC). All these independent entities ensured that the study protocol, governance, protections, and methods were ethical and appropriate. Furthermore, each participating core center needs to obtain approval from the respective Ethics Committee. Documentation of approval from each center is copied to the central governing office to ensure currency of approval is maintained.

All investigators (users) are mandated to sign the Investigator Declaration Form before obtaining credentials to use the registry. Both the project investigators and their institutions permit project-related monitoring, audits, and regulatory inspections, providing direct access to source data/documents. This may include, but is not limited to, review by HREC and institutional governance review bodies.

### Data protection

Proper handling of ethical, legal, social, and privacy issues must be a foundational component of the design, implementation, and long-term sustainability of a patient registry [7]. As part of the *retina.pt*, the IRD-PT module was designed to provide maximum data security and patient anonymity. Several well-defined procedures were put in place to protect individual patient data within the registry study. Data security, integrity, and availability is monitored and regulated.

All data transmissions between the user and the server are encrypted using 128-bit encryption (Secure Sockets Layer). The data are stored and backed up on secure servers at Portugal Telecom—Altice, TEAR 3 certified Datacenter. Anonymity of users is also closely guarded. Individual users can only see their own data. However, users may find other centers with included data on a specific disease and ask for research collaborations within the platform. Users can withdraw their data from the registry at any time, without providing a reason.

### Registry interface

Drop-down menus, pop-up explanatory notes, and tab-to-jump ensures rapid and user friendly data entry. Furthermore, *retina.pt* is a web-based application that is able to run on different server operating systems. Any device with Internet access and a recent browser can be used to interact with the application. Additional software on the user’s terminal is not required. When all mandatory fields have been filled, the User can “Finalize” the visit by pressing “Save”. The system has been designed in such a way that it will not allow a visit to be finalized unless all the mandatory fields have been filled and all numerical data fall within prespecified ranges. Additionally, the platform allows data to be automatically filled in by third party EHR applications with identically structured information, or the possibility of the user to generate a *pdf* document that can be printed/copied to the hospital EHR system. Moreover, storage and retrieval of clinical images is possible in the patient-specific page.

### Data quality

High quality data of rare diseases registries is considered to be one of the most important elements in the establishment and maintenance of a registry [20]. Quality assurance includes quality improvement activities such as medical, clinical, and record audit and observational studies, to which the ethical principles of research apply.

### Interoperability

Upon the development of the *retina.pt* platform, interoperability was a key issue. First, the registry has two available languages to choose from: Portuguese and English. Second, the age-related macular degeneration (AMD) module of *retina.pt* is already linked to the Fight Retinal Blindness! (FRB!) Project registry [2] and efforts are in place to connect it to the International Consortium for Health Outcomes Measurement (ICHOM) AMD registry. Third, the platform is serving as the eCRF for an upcoming post-market authorization clinical trial. Rare diseases are a prime example of a research area that can strongly profit from coordination on a European and international scale. To allow interoperability of the IRD-PT module with other IRD registries across the world, all the diseases are coded accordingly to ICD9, ICD10, ICD11, and ORDO (ORPHA codes) numbers. Furthermore, the genes are coded according to the OGG and MIM, and patient signs and symptoms are coded according to HPO. This is in accordance with the eye-specific dataset of the Clinical Patient Management System (CPMS) of the ERN-EYE [17]. Notably, ORDO, HPO, OGG and MIM are open-access, interoperable, community-driven, available in multiple languages and regularly updated.

## Data Availability

The datasets used and/or analyzed during the current study are available from the corresponding author on reasonable request.
